# Neuromelanin-Sensitive Magnetic Resonance Imaging in Schizophrenia: A Meta-Analysis of Case-Control Studies

**DOI:** 10.3389/fpsyt.2021.770282

**Published:** 2021-10-28

**Authors:** Lara Wieland, Sophie Fromm, Stefan Hetzer, Florian Schlagenhauf, Jakob Kaminski

**Affiliations:** ^1^Department of Psychiatry and Psychotherapy Charité Campus Mitte, Charité-Universitätsmedizin Berlin, Berlin, Germany; ^2^Department of Psychiatry and Psychotherapy Charité Campus Mitte, Charité-Universitätsmedizin Berlin, Einstein Center for Neurosciences Berlin, Berlin, Germany; ^3^Berlin Center for Advanced Neuroimaging, Charité-Universitätsmedizin Berlin, Berlin, Germany

**Keywords:** neuromelanin, biomarker, schizophrenia, quantitative MRI, psychosis

## Abstract

**Background:** Psychiatry is in urgent need of reliable biomarkers. Novel neuromelanin-sensitive magnetic resonance imaging (NM-MRI) sequences provide a time-efficient and non-invasive way to investigate the human brain *in-vivo*. This gives insight into the metabolites of dopaminergic signaling and may provide further evidence for potential dopaminergic alterations in patients with schizophrenia (SCZ). The present systematic review provides a meta-analysis of case-control studies using neuromelanin-sensitive sequences in SCZ vs. healthy controls (HC).

**Methods:** According to predefined search terms and inclusion criteria studies were extracted on PubMed. Meta-analyses with a fixed and random-effects model with inverse variance method, DerSimonian-Laird estimator for τ^2^, and Cohen's *d* were calculated. Bias was assessed using funnel plots. The primary study outcome was contrast-to-noise ratio (CNR) in the substantia nigra compared between HC and SCZ.

**Results:** The total sample of *k* = 6 studies included *n* = 183 cases and *n* = 162 controls. Across all studies we found a significant elevation of CNR in the substantia nigra (*d* = 0.42 [0.187; 0.655], *z* = 3.521, *p* < 0.001) in cases compared to controls. We found no significant difference in the control region of locus coeruleus (*d* = −0.07 [−0.446; 0.302], *z* = −0.192, *p* = 0.847), with CNR for the latter only reported in *k* = 3 studies.

**Conclusion:** CNR in the substantia nigra were significantly elevated in cases compared to controls. Our results support neuromelanin as a candidate biomarker for dopaminergic dysfunction in schizophrenia. Further studies need to assess this candidate marker in large, longitudinal cohorts and address potential effects of disease state, medication and correlations with symptoms.

## Introduction

For decades the search for novel biomarkers has driven neurobiological research on schizophrenia. Although there is no comprehensive mechanistic pathophysiological model to date, excessive dopaminergic signaling in the midbrain has been hypothesized to be central in the etiology of psychotic symptoms (1–3). The primarily used methods in molecular imaging, such as positron emission tomography (PET) or single photon emission computed tomography (SPECT) reliably found dopaminergic aberrations in patients with schizophrenia (4). Similarly, dopaminergic aberrations in patients with a diagnosis of bipolar disorder and psychotic symptoms were found (5). Therefore, dopaminergic signaling might function as a transdiagnostic marker for the symptomatically uniform category of psychosis instead of being limited to the diagnostic group of schizophrenia. However, obtaining these *in vivo* proxies of dopamine function is challenging in patient populations, since the needed radiopharmaceuticals render these measures highly expensive, invasive and time-consuming. Technological advances, such as improved signal intensity and increased signal-to-noise ratio recently enabled neuromelanin-sensitive magnetic resonance imaging (NM-MRI) as a possible readily available window into molecular alterations in psychiatric disorders *in vivo* (6, 7).

**Figure 1:**
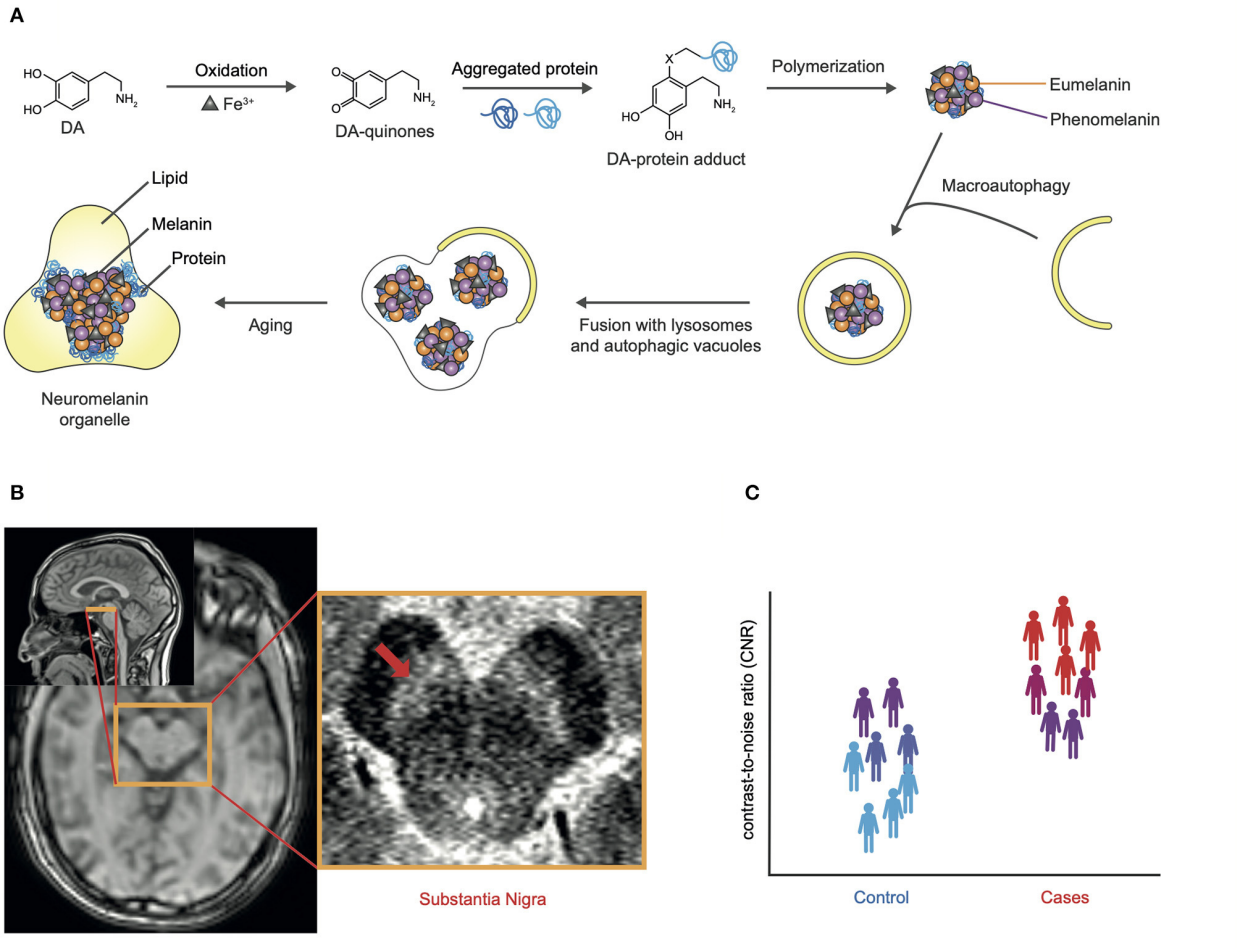
**(A)** Hypothesized mechanisms of NM biosynthesis adapted from Sulzer et al. (11): Dopamine (DA) oxidates, advanced by ferric iron (Fe^+3^). Oxidized DA-quinones react with cytosolic proteins and are polymerized, thereby initiating formation of a melanin-protein component with eumelanin and pheomelanin and resulting in an undegradable iron-melanin-protein complex. Macroautophagy is initiated and the complex is taken up into an autophagic vacuole. Fusion with lysosomes enables this vacuole to merge with other vacuoles, containing lipids and proteins and eventually result in neuromelanin organelles, that accumulate with increasing age of the organism. **(B)** NM-MRI of an exemplary slice lay-out (yellow) of the substantia nigra (SN) (red arrow) and its position within the brainstem on the left hand side localizer images. **(C)** Schematic scatter plot depicting contrast-to-noise ratio on the ordinate in controls (blue) and cases (red) with a dimensional biomarker approach (violet).

Neuromelanin (NM) is a byproduct of dopamine and catecholamine synthesis, primarily found in the dopaminergic neurons of the substantia nigra (SN) and the noradrenergic neurons of the locus ceruleus (LC) (8). It appears following several reactions, which begin with the oxidation of cytosolic dopamine, advanced by ferric iron (Fe^+3^). These oxidized dopamine-quinones react with cytosolic proteins and are polymerized, which results in an undegradable iron-melanin-protein complex. Possibly, because they are too large for proteasome degradation, macroautophagy is initiated and the complex is taken up into an autophagic vacuole. Fusion with lysosomes enables this vacuole to merge with other vacuoles, containing lipids and proteins and eventually result in neuromelanin organelles, that accumulate with increasing age of the organism (9, 10) (see Figure 1).

The specific features from the interaction between neuromelanin and iron in the iron-neuromelanin complexes make them highly paramagnetic and give them their unique signature in magnetic resonance imaging. A combination of reduced magnetization transfer and shortened T1 relaxation times results in high signal intensities for NM-containing brain regions (11). Although too small for conventional MRI techniques, novel NM-MR sequences acquired at a higher field-strength are sensitive enough to detect and quantify tissue containing neuromelanin. Initially, NM-MRI was developed as a candidate biomarker for Parkinson's disease, which is marked by the depletion of dopaminergic neurons in the SN and noradrenergic neurons in the LC (12). Typically, anatomical reference regions, which do not contain neuromelanin (such as crus cerebri) are used to calculate so-called contrast-to-noise ratios (CNR). In order to obtain these CNR, the signal intensity in the SN is set in ratio to the added signal intensities of the SN and a region without neuromelanin [see e.g., (12–15)], as the following exemplary formula shows:


$$\begin{array}{l} \text{CNR }=\frac{{\text{Neuromelanin}}_{target\;region}\;{\text{Neuromelanin}}_{reference\;region}}{{\text{Neuromelanin}}_{reference\;region}} \end{array}$$

After this initial breakthrough as a biomarker for neurodegenerative illness, a multitude of studies employed NM-MRI in patients with Parkinson's disease. Integrating these results in a recent meta-analysis, NM-MRI shows a pooled sensitivity of 89% and a pooled specificity of 83% in differentiating healthy individuals from those with Parkinson's disease (16). However, its usefulness as a biomarker beyond Parkinson's disease has been unclear thus far. As described above, dopaminergic alterations in the nigrostriatal and mesolimbic pathway have been hypothesized as a potential cause for psychosis (17), making the SN and ventral tegmental area (VTA) ideal candidate targets for NM-MRI. Due to the technical difficulties in detecting VTA with early NM-MRI sequences [e.g., (15)] the focus of the present review will be the SN.

In an extensive validation study, by Cassidy et al. (9), NM-MRI signal intensity was associated with regional NM concentration in post-mortem human brain tissue of the SN. In contrast to previous studies, a semiautomated voxelwise approach to identify SN and reference regions was used to avoid high inter-rater variability, present in manual tracing approaches. When investigating the relation to dopaminergic alterations, NM-MRI CNR were positively related to dopamine release in the dorsal striatum and resting blood flow in the SN in patients with schizophrenia. According to these results, NM-MRI in the SN could serve as a proxy of individual differences in presynaptic dopaminergic function.

Several studies have established excellent test-retest reliability with voxelwise intraclass coefficients (ICC) above 0.9 and test-retest intervals of several weeks in healthy individuals (18–20) and a few hours in a small sample of schizophrenic patients (9). This provides further evidence that NM-MRI might be a suitable marker with clinical value.

In order to elucidate the use of NM-MRI as a biomarker for schizophrenia, a quantitative synthesis of studies investigating NM-MR in case-control design is needed. To this end, all available case-control studies using NM-MRI in schizophrenia spectrum disorders are reviewed. We hypothesize that CNR of the NM-MRI signal in the SN will be significantly elevated in patients compared to controls, whereas CNR in the LC will not be significantly different between patients and controls. Furthermore, a meta-regression with year of publication and age as predictors will be calculated to control for further sources of variance.

## Methods

### Study Selection

This review was completed according to PRISMA (Preferred Reporting Items for Systematic Reviews and Meta-Analyses) guidelines (21). Inclusion criteria and methods for analyses were pre-specified and documented in a preregistered protocol {r} [https://osf.io/fykum] on January 6, 2021, publicly available on the OSF (Open Science Framework). PubMed was searched from inception to January 6, 2021, according to the following search algorithm: (“neuromelanin” OR “qMRI” OR “hMRI”) AND (MRI) AND (“schizophrenia” OR “schizophrenic” OR “psychosis”). A total of nine entries on PubMed and two additional studies, found via citation search, were screened. Studies were included according to the following predefined criteria: (1) case-control studies including patients with a diagnosis from the schizophrenia spectrum, (2) NM-MRI sequences were used and (3) they contained sufficient information on the outcome CNR. Studies or subsets of studies were excluded in the following case: (1) exclusively investigating other populations than patients with schizophrenia, (2) study designs only including patients without comparison to healthy controls (3) article using different outcome variables than CNR (22). Five studies with one study including two separate samples were found to be eligible (9, 13–15, 23) (see Table 1). Age and sex distribution were extracted for those studies. CNR values were obtained by the study authors in two cases (9, 13).

**Table 1:** Details of MR sequences and patient characteristics.

**References**	**Sample**	**Age of onset in patients, Mean (SD)**	**Reference region**	**ROI tracing**	**Scanner type**	**MR-sequence**	**TE (in ms)**	**TR (in ms)**
Cassidy et al. (9) (SCZ/CHR)	SCZ/HC/CHR	NA	Crus cerebri	Semi-automated	3TE GE	2D GRE-MT	2.68	260
Jalles et al. (13)	FEP/HC	NA	NA	Manual	3T Phillips	FSE	14	600
Shibata et al. (14)	SCZ/HC	26.5 (6.9)	SCP decussation, pontine tegmentum	Manual	3TE GE	FSE	14	600
Sasaki et al. (23)	SCZ/HC/MDD^a^	26.3 (6.8)	SCP decussation, pontine tegmentum	Manual	3TE GE	FSE	14	600
Watanabe et al. (15)	SCZ/HC	22.9 (10.1)	Midbrain tegmentum	Manual	3TE GE	3D-SPGR	2.4	38.4

*GRE, gradient response echo; FSE, fast spin echo; SPGR, spoiled gradient-recalled; SCP, superior cerebellar peduncle; ROI, region of interest; TE, time to echo; TR, time to repetition; SCZ, schizophrenic patients; FEP, first episode psychosis; CHR, clinical high-risk individuals; HC, healthy controls; MDD, major depressive disorder*.

a *MDD subgroup not included in present review*.

### Quality Assessment

The methodological quality of all eligible studies was evaluated by two authors independently, using the Newcastle– Ottawa Scale (NOS) for assessing the quality of non-randomized studies in meta-analysis (24).

The statistical software R (version 3.6.3; R Foundation for Statistical Computing, Vienna, Austria) with the meta package (25) for meta-analysis of effect size and with the meta for package (26) for meta-analysis of variance was used to analyze all data. The standard *p* < 0.05 criterion was applied to evaluate statistical significance from meta-analytical results. Outlier detection was applied as described in the supplement.

### Meta-Analysis

Novelty of the method and scarcity of eligible studies prevented an a priori estimate of heterogeneity. Therefore, a random-effects, as well as a fixed-effects model were calculated and compared. Hedge's g and DerSimonian-Laird estimator for τ were used for calculating pooled effect sizes while taking variance into account. Since there were only six studies available in total, no subgroup analyses were conducted, according to the standard minimum criterion of *k* = 10 studies (27). However, to account for potential differences between clinical high-risk (CHR) and SCZ groups we ran the meta-analysis both with and without the CHR sample in one of the studies (see Results section Meta-Analysis and supplement respectively).

### Meta-Regression

Meta-regression analyses for publication year and mean age across both groups were calculated to estimate the effect of moderating variables on effect sizes between studies. Hedge's g and DerSimonian-Laird estimator for τ were used for calculating pooled effect sizes while taking between-study variance into account.

### Meta-Analysis of Variance

In order to account for possibly increased variability in patient group and discover potential subgroups the variance ratios were calculated. The so-called variability ratio (VR) is the natural logarithm of the ratio of the two standard deviations of both groups. The coefficient of variation ratio (CVR) is the natural logarithm of the ratio between the coefficients of variation from two groups. It takes the interdependence of group mean and standard deviation into account (28). For more details on how to compute VR and CVR refer to the supplement.

## Results

### Study Selection

Nine studies were initially screened (as in PRISMA flow chart for study selection, see Supplementary Figure 1) by two of the authors independently. Authors of original studies were successfully contacted in two cases and contributed further data. Five studies were included, including 183 patients from the schizophrenia spectrum and 162 controls with patient sample sizes reaching from 20 to 52. Out of the included studies, one study (9) included two patient samples with respective matched control samples. Only the SCZ sample and not the CHR was included in the main text but for supplementary results treated as two separate studies, resulting in a total of six samples. Four studies reported data from medicated patients with schizophrenia (14, 15, 23) or a mix of medicated and unmedicated patients (9). One study reported data from medicated patients with first-episode psychosis (13) with substance abuse. One study reported data from clinical high-risk individuals (9). In all studies patients and controls were matched according to age and in five out of six studies patients and controls were matched according to sex (9, 14, 15, 23).

### Meta-Analysis

**Figure 2:**
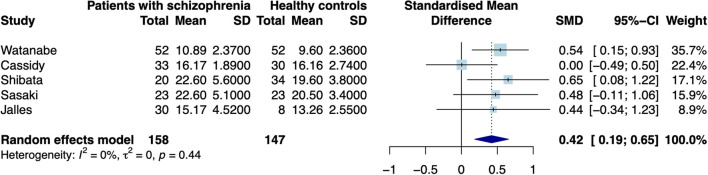
Forest plot of random effects analysis of mean group differences. Filled squares represent estimates per study and whiskers represent confidence intervals. Diamonds represent the point estimates with confidence intervals for an overall estimate in subgroups and the overall pooled effect across all studies.

**Figure 3:**
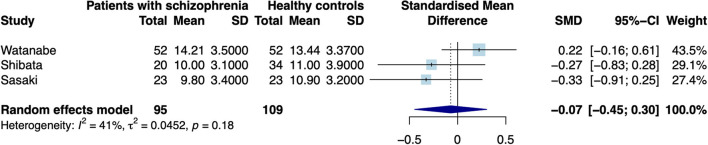
Forest plot of random effects analysis of mean group differences. Filled squares represent estimates per study and whiskers represent confidence intervals. Diamonds represent the point estimates with confidence intervals for an overall estimate in subgroups and the overall pooled effect across all studies.

When excluding the CHR sample (9), the random effects model revealed a significant increase in patients compared to controls in mean estimates for SN (*d* = 0.42 [0.187; 0.655], *z* = 3.521, *p* < 0.001, see Figure 2) and showed no significant difference for neuromelanin in the LC (*d* = −0.07 [−0.446; 0.302], *z* = −0.376, *p* = 0.706, see Figure 3). Including the CHR sample did lead to a slight decrease from *d* = 0.42 to *d* = 0.37 but did not change results fundamentally (see Supplementary Figure 9). Fixed effects modeling did also not change the results and are found in the supplement (see Supplementary Figures 7, 8 for forest plots and statistics).

The test for heterogeneity between studies showed a low amount of heterogeneity for CNR in the SN (τ^2^ = 0.00, I^2^= 4.28%), and a medium amount of heterogeneity in the LC (τ^2^ = 0.04, I^2^ = 40.78%) according to conventional standards (29). Formal testing for bias using funnel plot asymmetry (30) revealed no significant source of bias [see Supplementary Figure 2, t(4) = −0.40, *p* = 0.708]. Using trim-and-fill adjustment for bias no missing studies were added. *Post-hoc* power analyses revealed a power of 74.74%.

### Meta-Regression

A random-effects meta-regression was conducted to control for further sources of variance. Publication year showed a trendwise, albeit not significant moderating effect (QM = 3.26, *p* = 0.071, see Supplementary Figure 3). There was no significant moderating effect for age of the investigated subjects (QM = 1.60, *p* = 0.205, see Supplementary Figure 4).

### Meta-Analysis of Variance Ratio

The calculation of a random-effects model for differences in variability ratio revealed no significant effect in patients as compared to controls (logVR = 0.08 [−0.231; 0.388]; *z*=0.497; *p* = 0.619, see Supplementary Figure 5). Taking possible effects of mean differences into account, we calculated coefficient of variation ratio. The adjusted measure also shows no significant difference (logCVR = −0.01 [−0.276; 0.249]; *z* = −0.103; *p* = 0.918, see Supplementary Figure 6) across all studies.

## Discussion

We could show that neuromelanin-sensitive CNR in the SN were significantly higher in patients from the schizophrenia spectrum disorder, compared to healthy controls. Potential effects of age were not detectable in this meta-analysis. These results contribute to the development of NM-MR imaging of the SN as a promising biomarker for patients with schizophrenia.

However, some open questions remain regarding the relation to transdiagnostic features of the biomarker. The specificity of the signal, and the potential impact of confounds such as medication and age need to be addressed in future studies. To begin with, NM-MRI CNR were only related to positive symptom severity in clinical-high risk individuals as well as clinically manifest patient(s) with schizophrenia in one study (9). The absence of this association in previous studies (22, 23) might be explained by differential effects of antipsychotic medications. As suggested with regard to pharmacological imaging (5), a transdiagnostic perspective on biomarkers of psychosis is more useful than strict adherence to diagnostic categories. This assumption aligns with the positive association between symptom scores and NM-MRI signal intensity, reported in one study on a syndromal and prodromal sample (9). It also demonstrates the importance of assessing subjects, regarding a transdiagnostic psychotic phenotype in future studies.

Results regarding the anatomical specificity of this biomarker must be interpreted with caution, considering that CNR values for the LC were only available in three studies. Due to poor detection of VTA signals, findings are limited to the SN. However, given the more prominent findings concerning hyperdopaminergic alterations in associative regions (i.e., associative and sensorimotor striatum) (17), that mainly receive input from SN (31), this does not limit our findings. In a further study (22), which could not be included in the present meta-analysis due to different reporting of group differences as absolute instead of relative values, VTA values were significantly decreased in patients with schizophrenia. This supports the recently posed hypothesis of mesocortical deficits and nigrostriatal excess in the dopaminergic aberrations observed in schizophrenia (17). With regard to SN, one study used a different approach than the others and used a smaller subset of so-called psychosis overlap voxels instead of the whole SN (9). Furthermore, most studies included employed a manual tracing approach to determine regions of interest (ROI), which is less reliable compared to semiautomated thresholding techniques according to a recent systematic evaluation (19). Manual tracing is not only prone to human bias but also to circularity, when high-signal regions in the SN are determined to be ROI and subsequently used to calculate CNR (20). The voxelwise approach provides another clear advantage, in that it allows to investigate anatomical subregions of the midbrain and striatum, which are hypothesized to correspond to symptom groups of schizophrenia (32). Recently improved 3-D NM-MRI sequences also allow investigation of the VTA but were only investigated in patients with schizophrenia in a single study (22). The use of absolute values instead of CNR to determine signal intensity excluded the study from the present synthesis.

The present meta-analysis is limited by the following factors: The number of studies included is relatively low. However, NM-MR sequences have emerged within the last decades and were only recently suggested as a specific biomarker for schizophrenia (9). This is reflected in the relatively low number of studies in the field. Due to the low number of studies extracted, power was not sufficient for subgroup analyses, for example by clinical status. Another important possible confound is a potential effect of antipsychotic medication. Since there was only data on medication available in three out of six studies, there was too little data to provide a reliable statement. If the moderate effect sizes found in our meta-analysis are confirmed in further studies, usefulness of NM-MR sequences as a biomarker will have to be examined critically. One sample only includes patients with a comorbid substance use disorder (13), which has been shown to impact the dopaminergic system (33, 34) but also reflects clinical reality with a twofold increased use of substances in patients with first-episode psychosis (35) and therefore increases ecological validity of our meta-analysis.

Although test-retest reliability has been shown in 16 participants (9), larger samples investigating test-retest reliability and inter-site reliability of NM-MRI, particularly in psychotic samples, are warranted. Furthermore, as age effects are well-established in NM-MRI (11), potential interaction between diagnosis and age have to be addressed in future studies. Crucially, the translational application requires optimization of neuromelanin-sensitive sequences with short acquisition times (36, 37) and harmonization across sites.

## Data Availability Statement

Publicly available datasets were analyzed in this study. This data can be found at: https://github.com/agschlagenhauf/qMRI.

## Author Contributions

LW, SF, SH, FS, and JK: conceptualization, writing—review, and editing. LW and SF: data curation. LW, SF, and JK: formal analysis. LW: writing—original draft. All authors contributed to the article and approved the submitted version.

## Funding

This work was supported by the Einstein Center for Neurosciences (LW and SF), the German Research Foundation (Grant No. SCHL 1969/1-2/3-1/5-1) (FS) and the BIH-Charité Clinician Scientist Program funded by the Charité–Universitätsmedizin Berlin and the Berlin Institute of Health (JK).

## Conflict of Interest

The authors declare that the research was conducted in the absence of any commercial or financial relationships that could be construed as a potential conflict of interest.

## Publisher's Note

All claims expressed in this article are solely those of the authors and do not necessarily represent those of their affiliated organizations, or those of the publisher, the editors and the reviewers. Any product that may be evaluated in this article, or claim that may be made by its manufacturer, is not guaranteed or endorsed by the publisher.
