# Supplementary material for: Neuromelanin-Sensitive Magnetic Resonance Imaging in Schizophrenia: A Meta-Analysis of Case-Control Studies
Source: Front Psychiatry. 2021 Oct 28;12:770282. doi: 10.3389/fpsyt.2021.770282 (PMC8581671; doi:10.3389/fpsyt.2021.770282)
Supplement: Supplementary file 1 [file Data_Sheet_1.docx]

Supplementary material

**Neuromelanin-sensitive magnetic resonance imaging in schizophrenia: a meta-analysis of case-control studies.**

Wieland et al.

Study selection and data extraction

The selection process is reported according to preferred reporting items for systematic reviews and meta-analysis (PRISMA) guidelines (Moher et al., 2009). Selected studies are reported according to a prespecified protocol [<https://osf.io/fykum>] on January 6, 2021, publicly available on the OSF (Open Science Framework). During screening title and abstracts were scanned and case-control studies on patients with schizophrenia using neuromelanin-sensitive magnetic resonance imaging were included. Exclusion criteria were the lack of a control group and use of absolute signal intensity values instead of contrast-to-noise ratios (CNR). If there were analyses on separate samples reported in the same article, sample were treated as separate studies.

Study quality assessment

Study quality was assessed according to the Newcastle-Ottawa guidelines (Wells, G. A, Shea, B., O’Connel, 2009), as suggested by the US Agency for Healthcare Research and Quality. Selection of subjects and controls, comparability of cohorts and outcome measures are the main criteria for evaluating quality of the studies. Despite some concern over underspecified criteria (Stang, 2010) and limited interrater reliability (Hartling et al., 2013), it ultimately provides a readily applicable and useful guideline for basic evaluation of study quality. An additional appropriate criterion for comparability had to be chosen, in this case sex. Beyond rating all criteria, matching procedure of the original studies was confirmed, as described in the section below. Overall, we found that 5 out of 6 studies had good quality (see eTable 1).

Meta-analysis

Study effect sizes were weighted according to sample size. For estimating heterogeneity, the I^2^ value (according to conventional classification I^2^ values of 25%, 50% and 75% were taken as, low, moderate and high inconsistency respectively) (Higgins and Thompson, 2002). Publication bias was assessed by visual inspection of funnel plots and formally tested by Egger’s test for funnel plot asymmetry (Egger et al., 1997). This test statistic is based on a weighted linear regression of the standard error on the studies’ effect size. Due to low power of detecting funnel plot asymmetry a significance level of p<.1 for evaluating bias is recommended (Egger et al., 1997; Shi et al., 2019).

Because inspection of funnel plot asymmetry can be difficult, we additionally included analysis of possible effects of ‘file drawer studies’ by the trim-and-fill method (Duval and Tweedie, 2000). The results of Egger’s test showed no significant effect of bias (see results section in the manuscript). Trim and fill method revealed no missing studies (eFigure 6).

Outlier detection

Outlier detection was conducted by examining effect sizes of individual studies in comparison to the pooled effect size. If individual effect sizes were outside of the bounds of the confidence interval of the pooled effect size they were defined as outliers (Harrer et al., n.d.).

Meta-analysis of variance ratio

Possible heterogeneity in patient samples can be detected by using meta-analysis of variance ratio. One way to compute this variance is the so-called variability ratio (VR). According to the following formula, VR is the natural logarithm of the ratio of the standard deviations of patient and control group added to the sampling variance of the respective groups. The standard deviation is denoted by s and the respective sample by n:

Considering that higher mean values can be associated with higher variance (Taylor, 1961) a correction for potential bias in the variation ratio, namely the coefficient of variation ratio (CVR) was suggested (Nakagawa et al., 2015). It also takes the mean of the respective group into account:

Confirmation of matching
As described above study quality was evaluated according to the Newcastle-Ottawa guidelines (Wells et al., 2000). To alleviate potential concerns about comparability of studies the matching procedures for age and sex were verified for all studies. To compare the sex distribution in patients and controls -tests were calculated. The conventional p-value of p<0.05 was used for significance testing and for five out of six studies matching of patients and controls groups regarding sex was confirmed with non-significant results in X^2^ (3, N = 40) = 0.24, p = .62 (Cassidy et al., 2019), X^2^ (3, N = 71) = 0.65, p = .42 (Sasaki et al., 2010), X^2^ (3, N = 84) = 0.32, p = .57 (Shibata et al., 2008) and X^2^ (3, N = 104) = 0, p = 1 (Watanabe et al., 2014). The one study sample not matched on sex was matched on years of education (Jalles et al., 2020).

**PRISMA (2020) ABSTRACT CHECKLIST**

| **Topic** | **No.** | **Item** | **Reported?** |
| --- | --- | --- | --- |
| **TITLE** |  |  |  |
| **Title** | 1 | Identify the report as a systematic review. | Yes |
| **BACKGROUND** |  |  |  |
| **Objectives** | 2 | Provide an explicit statement of the main objective(s) or question(s) the review addresses. | Yes |
| **METHODS** |  |  |  |
| **Eligibility criteria** | 3 | Specify the inclusion and exclusion criteria for the review. | Yes |
| **Information sources** | 4 | Specify the information sources (e.g. databases, registers) used to identify studies and the date when each was last searched. | Yes |
| **Risk of bias** | 5 | Specify the methods used to assess risk of bias in the included studies. | Yes |
| **Synthesis of results** | 6 | Specify the methods used to present and synthesize results. | Yes |
| **RESULTS** |  |  |  |
| **Included studies** | 7 | Give the total number of included studies and participants and summarise relevant characteristics of studies. | Yes |
| **Synthesis of results** | 8 | Present results for main outcomes, preferably indicating the number of included studies and participants for each. If meta-analysis was done, report the summary estimate and confidence/credible interval. If comparing groups, indicate the direction of the effect (i.e. which group is favoured). | Yes |
| **DISCUSSION** |  |  |  |
| **Limitations of evidence** | 9 | Provide a brief summary of the limitations of the evidence included in the review (e.g. study risk of bias, inconsistency and imprecision). | Yes |
| **Interpretation** | 10 | Provide a general interpretation of the results and important implications. | Yes |
| **OTHER** |  |  |  |
| **Funding** | 11 | Specify the primary source of funding for the review. | Yes |
| **Registration** | 12 | Provide the register name and registration number. | Yes |

**PRISMA (2020) MAIN CHECKLIST**

| **Topic** | **No.** | **Item** | **Location where item is reported** |
| --- | --- | --- | --- |
| **TITLE** |  |  |  |
| **Title** | 1 | Identify the report as a systematic review. | Page 1 |
| **ABSTRACT** |  |  |  |
| **Abstract** | 2 | See the PRISMA 2020 for Abstracts checklist |  |
| **INTRODUCTION** |  |  |  |
| **Rationale** | 3 | Describe the rationale for the review in the context of existing knowledge. | Page 6 |
| **Objectives** | 4 | Provide an explicit statement of the objective(s) or question(s) the review addresses. | Page 6 |
| **METHODS** |  |  |  |
| **Eligibility criteria** | 5 | Specify the inclusion and exclusion criteria for the review and how studies were grouped for the syntheses. | Page 7 |
| **Information sources** | 6 | Specify all databases, registers, websites, organisations, reference lists and other sources searched or consulted to identify studies. Specify the date when each source was last searched or consulted. | Page 7 |
| **Search strategy** | 7 | Present the full search strategies for all databases, registers and websites, including any filters and limits used. | Page 7 |
| **Selection process** | 8 | Specify the methods used to decide whether a study met the inclusion criteria of the review, including how many reviewers screened each record and each report retrieved, whether they worked independently, and if applicable, details of automation tools used in the process. | Supplement |
| **Data collection process** | 9 | Specify the methods used to collect data from reports, including how many reviewers collected data from each report, whether they worked independently, any processes for obtaining or confirming data from study investigators, and if applicable, details of automation tools used in the process. | Supplement |
| **Data items** | 10a | List and define all outcomes for which data were sought. Specify whether all results that were compatible with each outcome domain in each study were sought (e.g. for all measures, time points, analyses), and if not, the methods used to decide which results to collect. | Page 7 |
|  | 10b | List and define all other variables for which data were sought (e.g. participant and intervention characteristics, funding sources). Describe any assumptions made about any missing or unclear information. | Page 7 |
| **Study risk of bias assessment** | 11 | Specify the methods used to assess risk of bias in the included studies, including details of the tool(s) used, how many reviewers assessed each study and whether they worked independently, and if applicable, details of automation tools used in the process. | Page 7 |
| **Effect measures** | 12 | Specify for each outcome the effect measure(s) (e.g. risk ratio, mean difference) used in the synthesis or presentation of results. | Page 8 |
| **Synthesis methods** | 13a | Describe the processes used to decide which studies were eligible for each synthesis (e.g. tabulating the study intervention characteristics and comparing against the planned groups for each synthesis (item 5)). | Supplement |
|  | 13b | Describe any methods required to prepare the data for presentation or synthesis, such as handling of missing summary statistics, or data conversions. | Page 7 |
|  | 13c | Describe any methods used to tabulate or visually display results of individual studies and syntheses. | Page 7 |
|  | 13d | Describe any methods used to synthesize results and provide a rationale for the choice(s). If meta-analysis was performed, describe the model(s), method(s) to identify the presence and extent of statistical heterogeneity, and software package(s) used. | Page 7 |
|  | 13e | Describe any methods used to explore possible causes of heterogeneity among study results (e.g. subgroup analysis, meta-regression). | Page 8 |
|  | 13f | Describe any sensitivity analyses conducted to assess robustness of the synthesized results. | Supplement |
| **Reporting bias assessment** | 14 | Describe any methods used to assess risk of bias due to missing results in a synthesis (arising from reporting biases). | Supplement |
| **Certainty assessment** | 15 | Describe any methods used to assess certainty (or confidence) in the body of evidence for an outcome. | Supplement |
| **RESULTS** |  |  |  |
| **Study selection** | 16a | Describe the results of the search and selection process, from the number of records identified in the search to the number of studies included in the review, ideally using a flow diagram. | Supplement |
|  | 16b | Cite studies that might appear to meet the inclusion criteria, but which were excluded, and explain why they were excluded. | Page 7 |
| **Study characteristics** | 17 | Cite each included study and present its characteristics. | Page 9 |
| **Risk of bias in studies** | 18 | Present assessments of risk of bias for each included study. | Supplement |
| **Results of individual studies** | 19 | For all outcomes, present, for each study: (a) summary statistics for each group (where appropriate) and (b) an effect estimate and its precision (e.g. confidence/credible interval), ideally using structured tables or plots. | Figure 2 |
| **Results of syntheses** | 20a | For each synthesis, briefly summarise the characteristics and risk of bias among contributing studies. | Supplement |
|  | 20b | Present results of all statistical syntheses conducted. If meta-analysis was done, present for each the summary estimate and its precision (e.g. confidence/credible interval) and measures of statistical heterogeneity. If comparing groups, describe the direction of the effect. | Page 13 |
|  | 20c | Present results of all investigations of possible causes of heterogeneity among study results. | Supplement |
|  | 20d | Present results of all sensitivity analyses conducted to assess the robustness of the synthesized results. | Supplement |
| **Reporting biases** | 21 | Present assessments of risk of bias due to missing results (arising from reporting biases) for each synthesis assessed. | Supplement |
| **Certainty of evidence** | 22 | Present assessments of certainty (or confidence) in the body of evidence for each outcome assessed. | Supplement |
| **DISCUSSION** |  |  |  |
| **Discussion** | 23a | Provide a general interpretation of the results in the context of other evidence. | Page 15 |
|  | 23b | Discuss any limitations of the evidence included in the review. | Page 15 |
|  | 23c | Discuss any limitations of the review processes used. | Page 16 |
|  | 23d | Discuss implications of the results for practice, policy, and future research. | Page 15/16 |
| **OTHER INFORMATION** |  |  |  |
| **Registration and protocol** | 24a | Provide registration information for the review, including register name and registration number, or state that the review was not registered. | Page 7 |
|  | 24b | Indicate where the review protocol can be accessed, or state that a protocol was not prepared. | Page 7 |
|  | 24c | Describe and explain any amendments to information provided at registration or in the protocol. | NA |
| **Support** | 25 | Describe sources of financial or non-financial support for the review, and the role of the funders or sponsors in the review. | Page 13 |
| **Competing interests** | 26 | Declare any competing interests of review authors. | Page 14 |
| **Availability of data, code and other materials** | 27 | Report which of the following are publicly available and where they can be found: template data collection forms; data extracted from included studies; data used for all analyses; analytic code; any other materials used in the review. | Supplement |


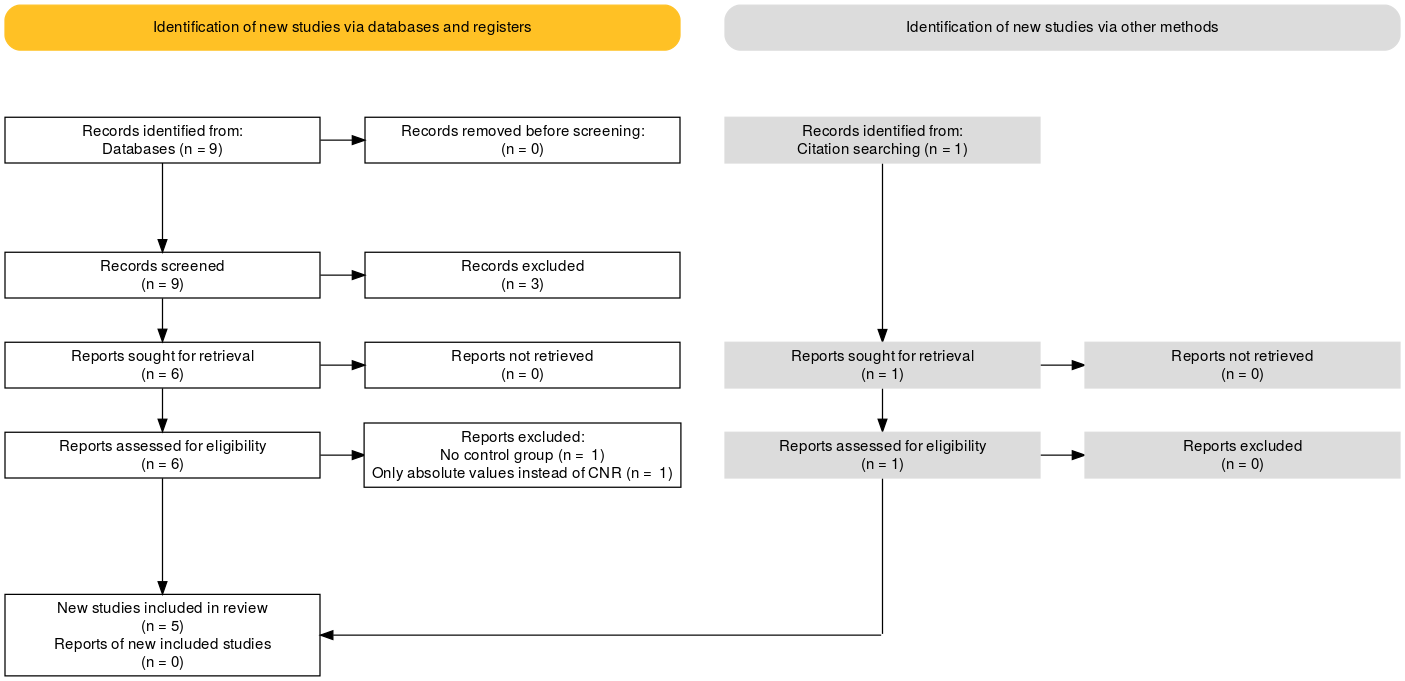


eFigure1 Flow chart of study selection according to preferred reporting items for systematic reviews and meta-analysis (PRISMA) guidelines.

eTable 1 Quality assessment of studies included in the meta-analysis based on the Newcastle Ottawa Scale.

|  | **selection** | | | |  | **comparability of cohorts** | |  | **outcome** | |  | **quality rating** | |
| --- | --- | --- | --- | --- | --- | --- | --- | --- | --- | --- | --- | --- | --- |
| **study** | adequate case definition | representativness  of cases | selection of controls | definition of controls |  | age | gender |  | secure | same in controls | **total score** |  |  |
| Cassidy et al. 2019 SCZ | * | * | * | * |  | * | * |  |  |  | 6 | good |  |
| Cassidy et al. 2019 CHR | * | * | * | * |  | * | * |  |  |  | 6 | good |  |
| Jalles et al. 2020 | * | * |  |  |  | * |  |  | * | * | 5 | fair |  |
| Shibata et al. 2012 | * | * |  |  |  | * | * |  | * | * | 6 | good |  |
| Sasaki et al. 2010 | * | * |  |  |  | * | * |  | * | * | 6 | good |  |
| Watanabe et al. 2014 | * | * | * | * |  | * | * |  | * | * | 8 | good |  |
| \| For converting the Newcastle Ottawa rating according to the scaling of the Agency for Healthcare Research and Quality standards we followed the following thresholds:  Good quality: 3 or 4 stars in selection domain AND 1 or 2 stars in comparability domain AND 1 or 2 stars in outcome domain  Fair quality: 2 stars in selection domain AND 1 or 2 stars in comparability domain AND 1 or 2 stars in outcome domain  Poor quality: 0 or 1 star in selection domain OR 0 stars in comparability domain OR 0 or 1 stars in outcome domain \| \| --- \| | | | | | | | | | | | | |  |

eFigure2 Funnel plot displaying standard error on the y-axis and standardized mean difference on the x-axis with estimated effects. No clear asymmetry as a possible source of bias can be detected. Formal testing with Egger’s test for funnel plot asymmetry shows no significant evidence for publication bias (t(4)=-0.40, p=.709).

eFigure3 Bubble plot of effect size on the y-axis and year of publication on the x-axis. Size of the bubbles are scaled with the size of the study and labelled with the respective first authors' name. Formal testing with a mixed effects model showed no significant moderating effect of year of publication year (beta=-0.05, Q=3.26, p=.071).

eFigure4 Bubble plot of effect size on the y-axis and mean age on the x-axis. Size of the bubbles are scaled with the size of the study and and labelled with the respective first authors' name.. Formal testing with a mixed effects model showed no significant moderating effect of mean age (beta=0.02, Q=1.60, p=.205).

eFigure5 Forest plot of random effects analysis of variability ratio comparing patient and control groups revealed no significant difference in variability ratio (logVR=0.08 [-0.231;0.388]; z=0.497; p=.619).

eFigure6 Forest plot of random effects analysis of coefficient of variability ratio (CVR) comparing patient and control groups revealed no significant difference in coefficient of variability ratio (logCVR=-0.01 [-0.276;0.249]; z=-0.103; p=.918).

eFigure7 Forest plot of fixed effects analysis of mean group differences comparing patient and control groups revealed a significant difference between patients and controls in mean estimates for neuromelanin content in the SN (d=0.42 [0.187; 0.655], z=3.521, p<.001).

eFigure8 Forest plot of fixed effects analysis of mean group differences comparing patient and control groups revealed no significant differences in mean estimates for neuromelanin in the LC (d=-0.03 [-0.305; 0.250], z=-0.192, p=.848).

eFigure9 Forest plot of fixed effects analysis of mean group differences comparing patient and control groups and including CHR group revealed a significant increase in patients compared to controls in mean estimates for SN (d=0.37 [0.143; 0.595], z=3.202, p=.001).

References

Cassidy, C.M., Zucca, F.A., Girgis, R.R., Baker, S.C., Weinstein, J.J., Sharp, M.E., Bellei, C., Valmadre, A., Vanegas, N., Kegeles, L.S., Brucato, G., Kang, U.J., Sulzer, D., Zecca, L., Abi-Dargham, A., Horga, G., 2019. Neuromelanin-sensitive MRI as a noninvasive proxy measure of dopamine function in the human brain. Proc. Natl. Acad. Sci. U. S. A. 116, 5108–5117. https://doi.org/10.1073/pnas.1807983116

Duval, S., Tweedie, R., 2000. Trim and Fill: A Simple Funnel-Plot-Based Method. Biometrics 56, 455–463.

Egger, M., Smith, G.D., Schneider, M., Minder, C., 1997. Bias in meta-analysis detected by a simple, graphical test. Br. Med. J. 315, 629–634. https://doi.org/10.1136/bmj.315.7109.629

Harrer, M., Cuijpers, P., Furukawa, T.A., Ebert, D.D., n.d. Doing Meta-Analysis in R: A Hands-on Guide. PROTECT Lab.

Hartling, L., Milne, A., Hamm, M.P., Vandermeer, B., Ansari, M., Tsertsvadze, A., Dryden, D.M., 2013. Testing the Newcastle Ottawa Scale showed low reliability between individual reviewers. J. Clin. Epidemiol. 66, 982–993. https://doi.org/10.1016/j.jclinepi.2013.03.003

Higgins, J.P.T., Thompson, S.G., 2002. Quantifying heterogeneity in a meta-analysis. Stat. Med. 21, 1539–1558. https://doi.org/10.1002/sim.1186

Jalles, C., Chendo, I., Levy, P., Reimão, S., 2020. Neuromelanin changes in first episode psychosis with substance abuse. Schizophr. Res. 220, 283–284. https://doi.org/10.1016/j.schres.2020.03.034

Moher, D., Liberati, A., Tetzlaff, J., Altman, D.G., Altman, D., Antes, G., Atkins, D., Barbour, V., Barrowman, N., Berlin, J.A., Clark, J., Clarke, M., Cook, D., D’Amico, R., Deeks, J.J., Devereaux, P.J., Dickersin, K., Egger, M., Ernst, E., Gøtzsche, P.C., Grimshaw, J., Guyatt, G., Higgins, J., Ioannidis, J.P.A., Kleijnen, J., Lang, T., Magrini, N., McNamee, D., Moja, L., Mulrow, C., Napoli, M., Oxman, A., Pham, B., Rennie, D., Sampson, M., Schulz, K.F., Shekelle, P.G., Tovey, D., Tugwell, P., 2009. Preferred reporting items for systematic reviews and meta-analyses: The PRISMA statement. PLoS Med. https://doi.org/10.1371/journal.pmed.1000097

Nakagawa, S., Poulin, R., Mengersen, K., Reinhold, K., Engqvist, L., Lagisz, M., Senior, A.M., 2015. Meta-analysis of variation: Ecological and evolutionary applications and beyond. Methods Ecol. Evol. 6, 143–152. https://doi.org/10.1111/2041-210X.12309

Sasaki, M., Shibata, E., Ohtsuka, K., Endoh, J., Kudo, K., Narumi, S., Sakai, A., 2010. Visual discrimination among patients with depression and schizophrenia and healthy individuals using semiquantitative color-coded fast spin-echo T1-weighted magnetic resonance imaging. Neuroradiology 52, 83–89. https://doi.org/10.1007/s00234-009-0595-7

Shi, L., Lin, L., Omboni, S., 2019. The trim-and-fill method for publication bias: Practical guidelines and recommendations based on a large database of meta-analyses. Med. (United States) 98. https://doi.org/10.1097/MD.0000000000015987

Shibata, E., Sasaki, M., Tohyama, K., Otsuka, K., Endoh, J., Terayama, Y., Sakai, A., 2008. Use of Neuromelanin-Sensitive MRI to Distinguish Schizophrenic and Depressive Patients and Healthy Individuals Based on Signal Alterations in the Substantia Nigra and Locus Ceruleus. Biol. Psychiatry 64, 401–406. https://doi.org/10.1016/j.biopsych.2008.03.021

Stang, A., 2010. Critical evaluation of the Newcastle-Ottawa scale for the assessment of the quality of nonrandomized studies in meta-analyses. Eur. J. Epidemiol. 25, 603–605. https://doi.org/10.1007/s10654-010-9491-z

Taylor, L.R., 1961. Aggregation, Variance and the Mean. Nature 189, 732–735. https://doi.org/10.1038/189732a0

Watanabe, Y., Tanaka, H., Tsukabe, A., Kunitomi, Y., Nishizawa, M., Hashimoto, R., Yamamori, H., Fujimoto, M., Fukunaga, M., Tomiyama, N., 2014. Neuromelanin magnetic resonance imaging reveals increased dopaminergic neuron activity in the substantia nigra of patients with schizophrenia. PLoS One 9, 1–6. https://doi.org/10.1371/journal.pone.0104619

Wells, G., Shea, B., D O’Connell, J., Peterson, V., Welch, M., Losos, Tugwell, P., 2000. The Newcastle–Ottawa Scale (NOS) for Assessing the Quality of Non-Randomized Studies in Meta-Analysis.
